# Beyond Beer‐Lambert Linear Regression: Multi‐Layer Modeling for Wide‐Range Concentration Quantification Using Ultraviolet‐visible Spectroscopy

**DOI:** 10.1002/ansa.70078

**Published:** 2026-04-02

**Authors:** Muhammed ALjifri, Carter Miller, Yanjun Qian, Ye Chen, Mo Jiang

**Affiliations:** ^1^ Department of Mathematics, College of Science & Arts King Abdulaziz University Rabigh Saudi Arabia; ^2^ Department of Chemical & Life Science Engineering Virginia Commonwealth University Richmond Virginia USA; ^3^ Department of Statistical Sciences and Operations Research Virginia Commonwealth University Richmond Virginia USA; ^4^ Schmidthorst College of Business Bowling Green State University Bowling Green Ohio USA; ^5^ The Polytechnic School Arizona State University Mesa Arizona USA

**Keywords:** chemometrics, global nonlinearity, local model, metal ion detection, partial least squares

## Abstract

Ultraviolet–visible (UV‐Vis) spectroscopy is widely used for chemical quantification due to its simplicity and low cost; however, accurate concentration prediction becomes challenging when target concentrations span wide ranges, where the global linearity assumption of the Beer‐Lambert law often breaks down. To address this limitation, we propose a multi‐layer modeling framework that exploits local linearity rather than relying on a single global model. Two approaches, dynamical‐layer regression and classified‐layer regression, are both integrated with principal component regression and partial least squares. The framework is evaluated using UV‐Vis spectra of nickel sulfate and cobalt sulfate solutions across concentrations ranging from 10^−^^6^ to 0.9 mol L^−^^1^, as well as mixed‐solution scenarios. The proposed methods consistently outperform conventional single‐layer linear models and global nonlinear models, achieving up to a 50% reduction in root mean squared error and *R^2^* values exceeding 0.99 for single‐solute datasets. These results demonstrate that the proposed framework provides a robust and effective solution for wide‐range concentration prediction in spectroscopic analysis.

## 1 Introduction

Ultraviolet–visible (UV‐Vis) spectroscopy, a non‐destructive analytical technique with simple operation, low cost, and rapid measurement [1, 2], has been widely accepted for identifying metal ions and monitoring their concentration change in industrial, environmental, and materials‐related applications [3, 4]. There is growing interest in accurately quantifying concentrations in practical situations (e.g., in mixtures and/or at non‐ideal concentrations) from UV‐Vis absorbance data, such as by developing advanced statistical models. A fundamental challenge in such data analysis lies in the high dimensionality and strong multicollinearity of spectroscopic measurements. Absorbance values are recorded at a wide range of wavelengths (e.g., 200 to 750 nm), many of which are highly correlated, making conventional regression methods unstable or ineffective. For similar issues with other chemicals (e.g., in food authentication [7, 8], water quality monitoring [9], and pharmaceutical or materials analysis [10, 11]), dimension‐reduction‐based methods such as principal component regression (PCR) [5] and partial least squares (PLS) [6] have been successfully applied. PCR reduces dimensionality by projecting spectral variables onto a smaller set of orthogonal components, whereas PLS extracts latent variables that maximize the covariance between spectral data and the response. They can also be integrated with other statistical methods, such as moving window PLS for improved local calibration [12] and independent component analysis for spectral separation in mixtures [13].

Despite their success, PCR and PLS rely on the assumption of a linear relationship between absorbance and concentration. This assumption originates from the Beer‐Lambert law, which states that monochromatic UV‐Vis absorbance (approximately single wavelength) is proportional to the chemical concentration in solution for a limited concentration range (typically within an order of magnitude between smaller and larger values) [14]. However, in practical UV‐Vis measurements, this linearity often breaks down, particularly in practical situations when concentration ranges span several orders of magnitude and more than one ion is present in solution with overlapping UV‐Vis absorbance.

Several factors contribute to deviations from the linearity of the Beer‐Lambert law, even for a single ion type. At low concentrations, mechanical and instrumental factors, such as detector limitations, wavelength‐dependent noise, and spectrometer response characteristics, can distort absorbance measurements. At high concentrations, chemical factors related to the analyte, including ion‐ion interactions, aggregation, ionization, or partial decomposition, may further alter absorption behavior at different concentration levels [15, 16, 17]. Small variations in the optical path length of the sample cell can also affect measured absorbance. These effects, as well as possible spectra overlapping among ions, collectively introduce nonlinearity into the relationship between UV‐Vis spectra and metal ion concentration.

To address the nonlinearity issue, several strategies have been tested with limited success. Some studies discretize concentration values into categorical classes, enabling classification‐based analysis but limiting precise quantitative prediction [18]. Others apply transformations, such as logarithmic scaling, to compress concentration ranges and improve model performance [19]. Nonlinear models, including artificial neural networks [20], have also been explored for spectroscopic analysis [15, 16]. However, those models typically require large training datasets to achieve stable performance, and their accuracy often deteriorates when sample sizes are limited [21].

Therefore, there remains a strong need for models that can handle nonlinearity across wide concentration ranges and mixture with overlapping spectra while maintaining robustness for moderate‐sized datasets. This study addresses this challenge by proposing a **multi‐layer modeling framework** that exploits local linearity (inspired by the linearity of the Beer‐Lambert Law within a short concentration range) rather than trying to stretch for a single global model. Two variants are developed: (1) **dynamical‐layer regression**, which refines predictions using local regression models guided by an initial estimation, and (2) **classified‐layer regression**, which categorizes data into concentration‐based classes and applies class‐specific regression models. The full wavelength range (200–750 nm) of the absorbance data was used to construct the models towards higher accuracy.

The proposed framework is integrated with PCR and PLS and evaluated using UV‐Vis spectra of nickel sulfate, cobalt sulfate, and their mixture (overlapping spectra) at concentrations spanning six orders of magnitude. By combining the stability of linear regression with adaptive local refinement, this approach aims to improve prediction accuracy in concentration over conventional global models. The work also aims to explore a new way to bridge the gap between the theoretical linearity of the Beer‐Lambert law and the practical nonlinearity in spectral data.

## 2 Materials and Methods

### 2.1 Chemicals and Materials

Nickel sulfate hexahydrate (NiSO_4_·6H_2_O, >98%) and cobalt sulfate hexahydrate (CoSO_4_·6H_2_O, >98%) were obtained from Sigma‐Aldrich and used without further purification. All solutions were prepared using deionized (DI) water. A semi‐micro quartz cuvette (Fisher‐brand) with a 10 mm optical path length was used for all UV‐Vis measurements.

### 2.2 Instrumentation and Conditions

UV‐Vis spectra were obtained using a Model 440 UV‐Vis spectrophotometer (SI Photonics) equipped with deuterium and tungsten light sources for the ultraviolet and visible regions, respectively. The light source switches from deuterium to tungsten at 460 nm. Quartz cuvettes were used to enable measurements in the UV region and to minimize potential interference in UV absorbance measurements. A blank DI‐water spectrum was recorded as the baseline prior to sample measurements. Spectra were collected using the SI 400 software and stored as *.csv files.

### 2.3 Sample Preparation and Spectral Acquisition

Single‐solute solutions were prepared by dissolving NiSO_4_ ·6H_2_O or CoSO_4_ ·6H_2_O in DI water to obtain concentrations ranging from 10*^−^* ^6^ M to 0.9 M. A 1.0 M stock solution was prepared and diluted to 0.1 M, 0.2 M, …, 0.9 M, followed by serial dilutions by a factor of 10 down to 10*^−^* ^6^ M. Each solution was vortexed for approximately 15 s before the next dilution step.

For each concentration level, three independent samples were prepared. Each sample was measured three times, yielding nine spectra per concentration. In the analysis, the average of the three spectra obtained from the same sample was treated as one observation. If the three spectra from the same sample differed substantially, the corresponding observation was excluded.

### 2.4 Mixed‐Solution Design and Preparation

Mixed solutions containing both NiSO_4_ and CoSO_4_ were prepared based on Latin hypercube sampling (LHS) [22]. Specifically, 50 combinations of Ni and Co concentrations were generated over the range 10*^−^* ^6^ M to 0.1 M. For each ion, solutions were prepared using the same dilution procedure described above. For each mixture, measured volumes of the respective stock solutions were combined using volumetric pipettes, followed by the addition of DI water to achieve a final volume of 10 mL and the targeted concentrations. Each mixed sample was vortexed for approximately 15 s prior to spectral measurement. Spectra for mixed solutions were collected using the same protocol as for the single‐solute solutions, with triplicate samples and three measurements per sample.

### 2.5 Review of PCR and PLS

In a linear regression model with multiple predictor variables (in our case, the input spectra), the ordinary least squares (OLS) method can be used to minimize the sum of squared differences between the predicted values and the actual response (the concentration of metal ions). However, when the number of predictors exceeds the number of observations, OLS estimation often fails [23]. In such cases, statistical techniques with dimension reduction, such as PCR and PLS, are generally employed. PCR entails projecting predictor variables onto a set of uncorrelated variables, known as principal components. These components, which represent linear combinations of the original predictors, are used in a linear regression model to predict the response variables. By reducing data dimensionality and selecting a subset of principal components that capture key information, PCR can enhance model accuracy and address concerns about multicollinearity, especially in the presence of numerous correlated predictors [23]. On the other hand, PLS constructs latent variables through linear combinations of original predictors that explain maximum variation in the response variable. Similar to PCR, PLS proves advantageous when the number of predictors surpasses the number of observations, making it a valuable tool in scenarios characterized by substantial predictor dimensions. Moreover, the PLS algorithm identifies components that maximize not only the variance explained in the predictors but also their correlation with the responses. By balancing the importance of predictors and responses, PLS effectively models their underlying relationship.

### 2.6 The Multi‐Layer Framework

Let **X** represent the input spectra and *y* the corresponding concentration of the target chemical. The dataset is divided into a training set $X_{\mathrm{tr}},y_{\mathrm{tr}}$ and a testing set $X_{\mathrm{ts}},y_{\mathrm{ts}}$. In a single‐layer approach, a regression model is trained using the entire training set $X_{\mathrm{tr}},y_{\mathrm{tr}}$ and then applied to $X_{\mathrm{ts}}$. To improve prediction performance in the presence of nonlinearity across wide concentration ranges, we propose a multi‐layer framework that uses an initial estimate to select a local subset of training data for refinement. Specifically, the first layer fits a global model using $X_{\mathrm{tr}},y_{\mathrm{tr}}$ to obtain an initial estimate $\hat{y}$ for each test sample. A second‐layer model is then trained using only the training data in the neighborhood of $\hat{y}$ and used to produce the final prediction. Since y is univariate, selecting the local training subset based on proximity in y‐space is computationally efficient.

**FIGURE 1:**
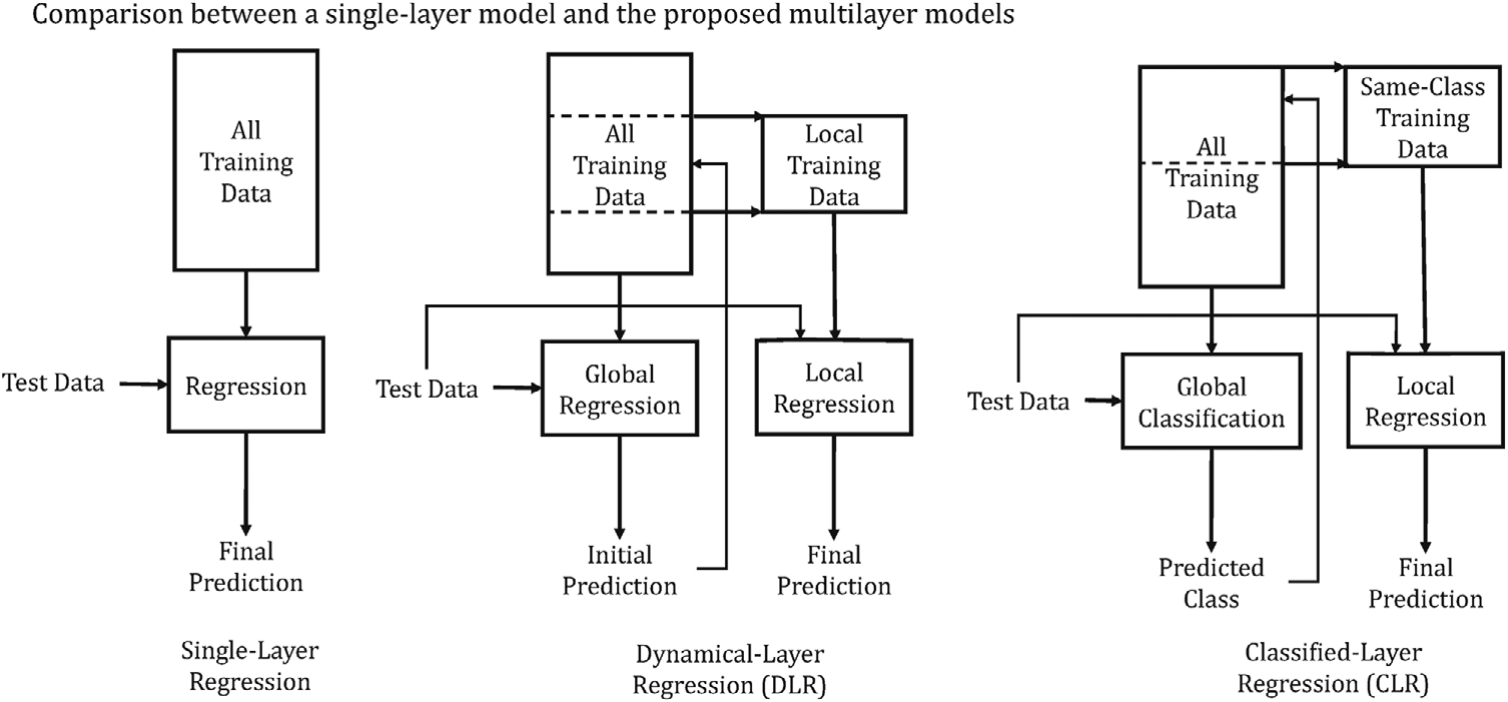
Schematic comparison of single‐layer regression (left), dynamical‐layer regression (DLR, middle), and classified‐layer regression (CLR, right). DLR refines an initial global estimate using a local training subset; CLR applies a class‐specific regression model based on a predicted concentration class.

We consider two strategies: (1) **dynamical‐layer regression** (DLR), which produces a quantitative estimate via a second‐layer regression model, and (2) **classified‐layer regression** (CLR), which assigns each test sample to a concentration class (e.g., low/high) and applies a class‐specific regression model. The DLR and CLR procedures, which are illustrated in Figure 1, are described below.

#### 2.6.1 DLR Method

We first fit a global regression model (e.g., PCR or PLS) using the entire $X_{\mathrm{tr}},y_{\mathrm{tr}}$ to obtain an initial estimate $\hat{y}$ *_ini_* for each test sample. DLR then defines a localized layer centered at $\hat{y}$ *_ini_* by selecting training samples whose concentrations fall within a specified interval. A second‐layer regression model is fitted on this local subset to obtain the final prediction. If the interval contains too few training samples to support a stable model, the initial estimate is retained. Algorithm 1 summarizes the DLR procedure.

Algorithm 1 **Dynamical‐layer regression (DLR).**

**Table:** 

**Step 1**: For each test sample, obtain an initial estimate $\hat{y}$ *_ini_* using the first layer regression model trained from the entire $X_{\mathrm{tr}},y_{\mathrm{tr}}$.
**Step 2**: Specify a scaling parameter *s* for controlling the width of the interval used to select local training data, where *s >* 1.
**Step 3**: Extract a subset of the training data $X_{R},y_{R}$, whose concentrations fall within the interval [$\hat{y}$ *_ini_ / s*, $\hat{y}$ *_ini_* · *s*].
**Step 4**: If $X_{R},y_{R}$ does not contain enough samples, then let the initial estimate be the final prediction, i.e., $\hat{y}$ *_fin_* = $\hat{y}$ *_ini_*. Otherwise, go to Step 5.
**Step 5**: Train the second‐layer regression model from the subset $X_{R},y_{R}$ and then obtain the final prediction $\hat{y}$ *_fin_* using the new model.

#### 2.6.2 CLR Method

The CLR method partitions the training data into concentration‐based classes. For example, when dividing the data into two classes, the median concentration can be used as the threshold. More generally, if the data are to be partitioned into C non‐overlapping intervals, then the (100×c/C)% quantiles can serve as the thresholds, where c=1,…,C−1. As the concentrations in the testing data are unknown, we utilize PLS discriminant analysis (PLS‐DA) [13] to classify each test sample into one of the predefined classes. We then fit a regression model within each class using the corresponding training data and apply the class‐specific model to obtain the final prediction. Algorithm 2 summarizes the CLR procedure for two classes (C=2).

Algorithm 2 **Classified‐layer regression (CLR).**

**Table:** 

**Step 1**: Find the median concentration of the training data, y *_med_*.
**Step 2**: Divide the training data into two classes, *H_tr_* and *L_tr_*, where *H_tr_* consists of training data with concentrations higher than y *_med_*, and *L_tr_* consists of training data with concentrations lower than or equal to y *_med_*.
**Step 3**: Train a regression model for each class separately: a high‐class model using *H_tr_* and a low‐class model using *L_tr_*.
**Step 4**: Fit a PLS‐DA classification model using the entire training set to learn the mapping from spectral data to classes.
**Step 5**: Apply the trained PLS‐DA model to predict the class (high or low) for each test sample.
**Step 6**: If a test sample is classified as high, use the high‐class regression model to obtain the final prediction ${\hat{y}}_{fin}$; otherwise, use the low‐class regression model.

### 2.7 Model Evaluation Metrics

In this study, we use the root mean squared error (RMSE) to assess the predictive performance of the proposed methods. The MSE is defined as the average of the squared differences between predicted and true values, and RMSE is obtained by taking its square root. RMSE measures the typical magnitude of prediction errors, with lower values indicating higher accuracy and higher values indicating poorer predictive performance [7, 24]. The RMSE is computed as:

$$\mathrm{RMSE}=\sqrt{\frac{1}{n_{ts}}\sum_{i=1}^{n_{ts}}{\left(y_{i}-{\hat{y}}_{i}\right)}^{2},}$$

where $y_{i}$ is the true concentration, ${\hat{y}}_{i}$ is the predicted value, and *n_ts_* is the number of samples in **X** *_ts_*. In addition to absolute error metrics, the coefficient of determination (*R^2^*) is commonly used in spectroscopic prediction to quantify the proportion of variance in the response explained by the model, which is given by:

$$R^{2}=1-\frac{\sum_{i=1}^{n_{ts}}{\left(y_{i}-{\hat{y}}_{i}\right)}^{2}}{\sum_{i=1}^{n_{ts}}{\left(y_{i}-\bar{y}\right)}^{2}},$$

where $\bar{y}=\sum_{i=1}^{n_{ts}}y_{i}/n_{ts}$ is the average of true concentration values in $y_{\mathrm{ts}}$. Note that *R^2^* ranges from 0 to 1, with values closer to 1 indicating better predictive performance.

To address the wide dynamic range in our dataset, we apply a base‐10 logarithmic transformation to the concentration values. Instead of predicting the original concentration $y_{i}$, we model $\log(y_{i})$ as the response variable. This transformation balances the influence of low and high concentrations, improving predictive performance across the entire range. Additionally, it compresses the response scale from (10^−6^, 10^−1^) to (−6, −1), which helps stabilize model training and enhance numerical efficiency. In the following context, unless otherwise noted, all predictions refer to the log‐transformed concentrations of Ni or Co ions from the single and mixed datasets, and performance metrics such as RMSE and *R^2^* are computed using $\log(y_{i})$ and $\log({\hat{y}}_{i})$ rather than the original values.

### 2.8 Parameter Tuning in the Multi‐Layer Framework

We perform parameter tuning to identify configurations that enhance generalization and reduce prediction error in the proposed multi‐layer framework. This process is conducted using systematic strategies such as K‐fold cross‐validation. For each candidate parameter setting, the model is trained on the training set and evaluated on a validation subset using error metrics such as RMSE. The parameter configuration that achieves the lowest validation error is then selected for final model evaluation on the test data.

#### 2.8.1 Cross‐Validation

Cross‐validation is widely used for parameter tuning in machine learning models. In K‐fold cross‐validation, the original dataset is randomly partitioned into K approximately equal subsets (or folds). Suppose the total number of samples is N. Then, each fold contains about N/K data points. The model is trained on K − 1 folds and validated on the remaining fold, and this process is repeated K times so that every data point is used once for validation and K − 1 times for training. Denote by RMSE*_j_* the RMSE of the model in the *j*th iteration of the cross‐validation process. Then, the overall average RMSE, denoted by $\bar{\mathrm{RMSE}}$, is given by

$$\bar{\mathrm{RMSE}}=\frac{1}{K}\;\sum_{j=1}^{K}\mathrm{RMS}E_{j}.$$

K‐fold cross‐validation, using $\bar{\mathrm{RMSE}}$ as the evaluation metric, provides a robust assessment of the model's generalization performance across different subsets of the data, thereby mitigating the risks of overfitting and underfitting. We adopt K‐fold cross‐validation because it offers a balanced tradeoff between computational cost and reliability of error estimation. In contrast to leave‐one‐out cross‐validation, it provides more stable error estimation with significantly lower computational burden. Moreover, compared to a single training/testing split, it provides improved robustness and consistency, making it particularly well‐suited for moderate‐sized datasets such as ours.

#### 2.8.2 Parameter Tuning in PCR/PLS

In both PCR and PLS regression models, the proper determination of the number of components is critical for ensuring predictive accuracy and generalization performance. An insufficient number of components may cause underfitting due to discarding essential spectral information, whereas an excessive number of components may capture noise and multicollinearity, resulting in overfitting [19, 25]. To balance these trade‐offs, we apply cross‐validation at both the first and second layers of the proposed framework. Specifically, the optimal number of components is selected as the one that minimizes $\bar{\mathrm{RMSE}}$ on the validation folds.

#### 2.8.3 Parameter Tuning in DLR/CLR

In the DLR method, the scaling parameter s determines the width of the interval used to select local training subsets for the second‐layer regression. Selecting an appropriate value of s is critical to balancing the inclusion of sufficient relevant data against the risk of incorporating irrelevant observations. A large s may result in a wide interval that may encompass too much of the dataset, diminishing the advantage of local refinement and effectively reverting to the global model. Conversely, a small s may yield a narrow interval that bears the risk of excluding informative samples near the boundary, thereby reducing prediction accuracy. To address this trade‐off, we systematically evaluate each candidate value in $s=10^{\ell }$, where ℓ = 0.1, 0.2, …, 3.0, and select the one that optimizes predictive performance while maintaining an appropriate balance between locality and data coverage.

In the CLR method, the number of classes C is a critical parameter that determines how the training data are partitioned. This partitioning directly affects the performance of the classification model and the accuracy of subsequent regression models, particularly when the response variable spans a wide range. To investigate the impact of C, we evaluate multiple class configurations and compare their predictive performance. This analysis enables us to quantify the sensitivity of CLR to the choice of C=1,2,…,6, and to identify the value that yields the highest predictive accuracy.

## 3 Results and Discussion

### 3.1 Spectral Characteristics and Data Distribution

**FIGURE 2:**
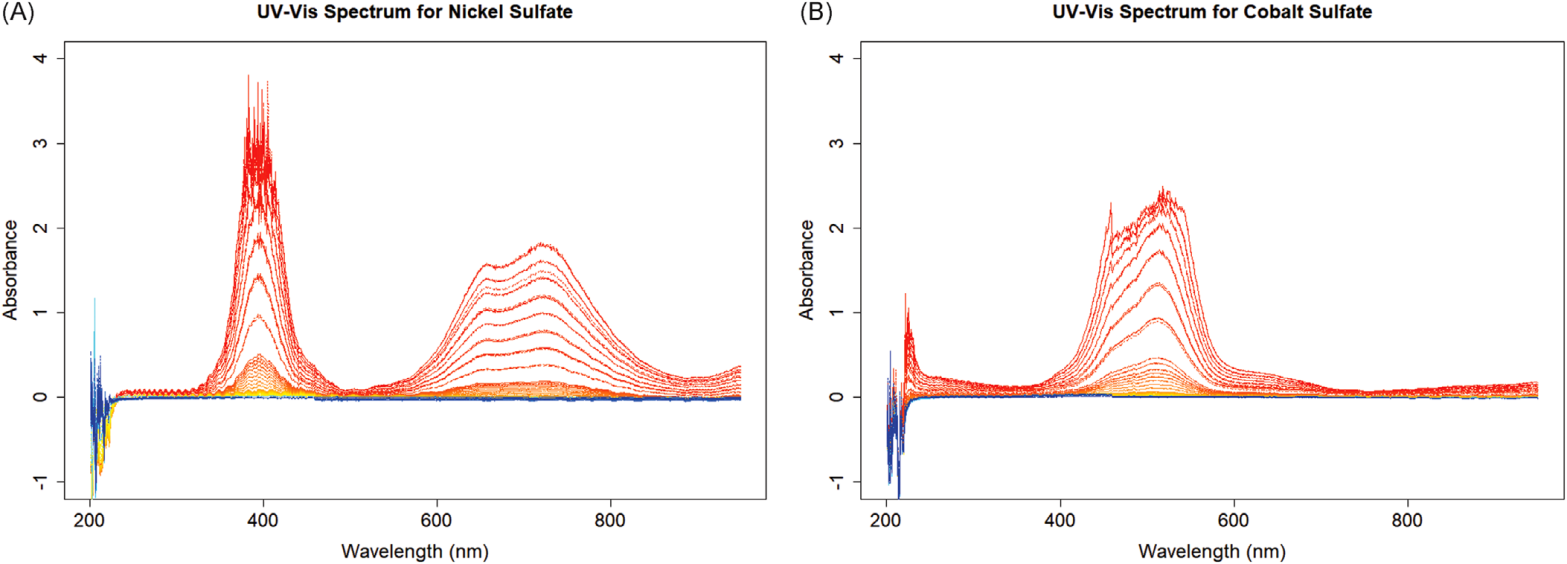
Ultraviolet–visible (UV‐Vis) absorbance spectra of single‐chemical (A) NiSO_4_ and (B) CoSO_4_ solutions. Spectra are color‐coded from red (high concentration, 0.9 M) to blue (low concentration, 10^−^^6^ M). Absorption peaks occur near 400 and 700 nm for Ni and 500 nm for Co.

Figure 2 illustrates the collected spectral datasets of NiSO_4_ and CoSO_4_, with each spectrum color‐coded from red to blue to represent decreasing concentrations of the corresponding chemicals. From their plots, we find that the Beer‐Lambert law holds near the corresponding peak wavelengths, approximately 400 and 700 nm for Ni and 500 nm for Co. However, when concentrations become too high or too low, the assumption of linearity may be violated due to potential measurement limitations and inherent uncertainty in the prepared samples. Additionally, the low‐wavelength (high‐frequency) parts of the UV‐Vis spectrum exhibit significant noise, displaying negative absorbance and rapidly changing values.

**FIGURE 3:**
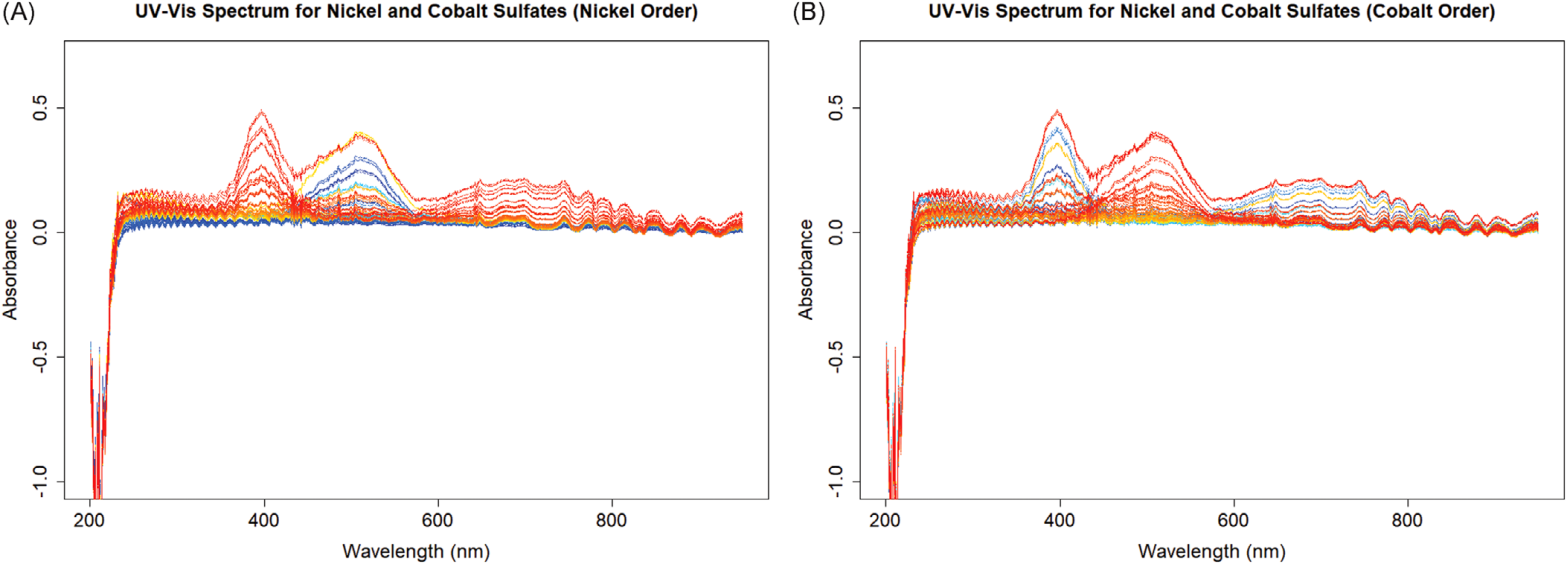
Ultraviolet–visible (UV‐Vis) absorbance spectra of mixed NiSO_4_ and CoSO_4_ solutions, color‐coded from red to blue by decreasing (A) Ni and (B) Co concentration. Spectral interference between the two ions is visible near 450 and 600 nm.

Figure 3 illustrates the mixed spectra dataset with NiSO_4_ and CoSO_4_ together, where the left plot is coded from red to blue based on the decrease of the Ni concentration, and the right plot is coded the same way based on Co concentration. The linear pattern near the peak wavelengths of the corresponding ions can still be observed, but there are more fluctuations in the data. The two kinds of ions interfere with each other near the wavelengths 450 nm and 600 nm. There are still apparent noises in the low‐wavelength parts of the spectra.

Figure S1 illustrates the distributions of Ni and Co concentrations in both the single‐solute and mixed solution datasets, where the red pluses indicate the Ni concentration in the NiSO_4_ solution, the purple crosses indicate the Co concentration in the CoSO_4_ solution, and the blue circles give both concentrations in the mixed dataset. Due to the wide concentration range from 10^−6^ M to 0.9 M, both axes are plotted on a logarithmic scale. For the single‐solute solutions, we collect a dense sample set of ion concentrations of the form $k\cdot 10^{m}$ M, where k ∈ {1, …, 9} and m ∈ {−6, …, −1}. For the mixed solutions, we generate a sparse and nearly uniform sample set of combinations of two ions, ranging from 10^−6^ M to 10^−1^ M using LHS.

### 3.2 Single‐solute Prediction Performance for the NiSO_4_ Dataset

We first evaluate the proposed multi‐layer model on the NiSO_4_ dataset. To construct training and testing sets, we randomly assign 70% of the data points to train the model and reserve the remaining 30% for testing. Note that our dataset contains multiple measurements for the same concentration, whose UV‐Vis spectra are nearly identical. Thus, we assign all observations with the same concentration to either the training or testing set, guaranteeing a more reliable assessment of model performance.

The parameter tuning details of DLR and CLR are shown in Figure S2 and Table S1. Figure S2 indicates that tuning s and C is critical to the performance of DLR and CLR, respectively. Specifically, PCR and PLS achieve the best performance when s is between 5 and 10 in the DLR, and when C=4 in the CLR.

**FIGURE 4:**
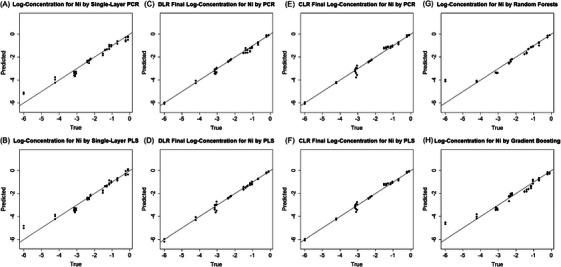
Predicted versus true log‐concentrations (mol L^−^^1^) for the NiSO_4_ test set. Subpanels: (A) single‐layer principal component regression (PCR), (B) single‐layer partial least squares (PLS), (C) dynamical‐layer regression (DLR) PCR, (D) DLR PLS, (E) classified‐layer regression (CLR) PCR, (F) CLR PLS, (G) random forests, and (H) gradient boosting. The diagonal represents perfect prediction.

Table 1 demonstrates the improvements achieved by DLR and CLR with optimal parameter settings compared to classic single‐layer PCR and PLS. For both PCR and PLS, DLR reduces the RMSE by half, from 0.29 to 0.15 for PCR and from 0.31 to 0.15 for PLS, and increases *R^2^* from 0.95 to 0.99. CLR achieves slightly higher RMSEs, ranging from 0.16 to 0.18, indicating its lower performance than DLR. Figure 4 presents scatter plots comparing the true and predicted log‐concentrations for NiSO_4_ across different models. Both multi‐layer methods notably improve predictions at the high and low ends of the log‐concentration range, where nonlinearity is most pronounced. Predictions in the intermediate range are also slightly improved, demonstrating that the proposed multi‐layer framework can train more accurate local models across the concentration levels. Compared with DLR, a key limitation of CLR is its reduced accuracy near class boundaries.

**TABLE 1:** Root mean squared error (RMSE) and *R^2^* comparison across single‐layer PCR/PLS, multi‐layer PCR/PLS (DLR and CLR), random forests (RFs), gradient boosting machines (GBMs), and artificial neural networks (ANNs) for NiSO_4_ log‐concentration prediction.

Method	PCR (Single)	PCR (DLR)	PCR (CLR)	PLS (Single)	PLS (DLR)	PLS (CLR)	RFs	GBM	ANNs
RMSE	0.2912	0.1534	0.1852	0.3097	0.1463	0.1625	0.5452	0.3462	3.004
*R^2^*	0.9583	0.9882	0.9828	0.9518	0.9892	0.9867	0.8688	0.9471	Negative

We also implement popular nonlinear machine learning models, including random forests (RFs) [26], gradient boosting machines (GBMs) [27], and artificial neural networks (ANNs) [20], and compare their performance with single‐ and multi‐layer linear models (PCR and PLS) in Table 1. Due to the high dimensionality of the spectral data and the relatively small training sample size, the ANNs model consistently predicts zero, resulting in a large RMSE and a negative *R^2^*. RFs and GBMs also exhibit lower prediction accuracy than single‐layer PCR and PLS. These results demonstrate that global nonlinear models are unable to adequately capture nonlinearity when the training dataset is of moderate size. In conclusion, the proposed multi‐layer method with PCR/PLS achieves the best performance for the NiSO_4_ dataset.

### 3.3 Single‐solute Prediction Performance for the CoSO_4_ Dataset

We apply the same analysis to the CoSO_4_ dataset. As in the previous section, we randomly select 70% of the data to train the model and evaluate RMSE on the remaining 30% used for testing. The parameter tuning details for the CoSO_4_ dataset are shown in Figure S3 and Table S2. The results indicate that the optimal s values of DLR (approximately 500 for PLS, 800 for PCR) are substantially larger than those obtained for the NiSO_4_ dataset, while larger values of C=5 of CLR are also preferred. This indicates that the tuning of s is highly sensitive to the characteristics of each dataset.

**FIGURE 5:**
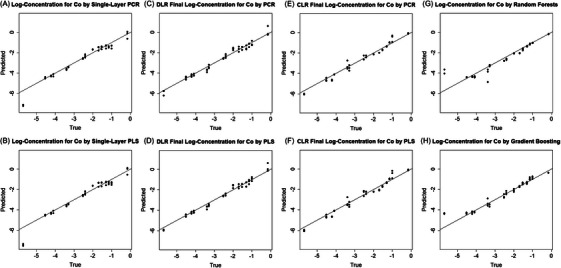
Predicted versus true log‐concentrations (mol L^−^^1^) for the CoSO_4_ test set. Subpanels: (A) single‐layer principal component regression (PCR), (B) single‐layer partial least squares (PLS), (C) dynamical‐layer regression (DLR) PCR, (D) DLR PLS, (E) classified‐layer regression (CLR) PCR, (F) CLR PLS, (G) random forests, and (H) gradient boosting. The diagonal represents perfect prediction.

The RMSE and *R^2^* values for DLR and CLR with optimal parameter configurations in Table 2 show that the most substantial improvements are again achieved by DLR, which reduces the RMSE from 0.43 to 0.24 for PCR and from 0.49 to 0.24 for PLS. *R^2^* values also increase to 0.97 for both PLS/PCR. CLR achieves moderate improvements, reducing the RMSE to 0.29/0.27 for PLS/PCR, but remains slightly inferior to DLR. Scatter plots in Figure 5 of predicted versus true log‐concentrations confirm that both multi‐layer methods improve predictions at the high and low ends of the concentration range, with modest enhancements in the intermediate range. The multi‐layer PCR/PLS also outperforms the three global nonlinear methods, i.e., RFs, GBMs, and ANNs, for the CoSO_4_ dataset.

**TABLE 2:** Root mean squared error (RMSE) and *R^2^* comparison across single‐layer PCR/PLS, multi‐layer PCR/PLS (DLR and CLR), random forests (RFs), gradient boosting machines (GBMs), and artificial neural networks (ANNs) for CoSO_4_ log‐concentration prediction.

Method	PCR (Single)	PCR (DLR)	PCR (CLR)	PLS (Single)	PLS (DLR)	PLS (CLR)	RFs	GBMs	ANNs
RMSE	0.4304	0.2430	0.2686	0.4941	0.2382	0.2888	0.5429	0.3911	3.004
*R^2^*	0.9183	0.9739	0.9682	0.8923	0.9750	0.9632	0.8699	0.9325	Negative

### 3.4 Mixed‐solution Prediction Performance and Limitations

Compared to the single‐chemical case, the mixed‐dataset introduces additional challenges due to overlapping spectral features. In this section, we evaluate the proposed framework for predicting the log‐concentration of Ni and Co ions using the mixed NiSO_4_ and CoSO_4_ datasets. To build the machine learning models for Ni or Co prediction, we combine 50% of the mixed dataset with the corresponding single chemical dataset for training. The remaining 50% of the mixed dataset is reserved for testing. We drop the ANN model due to its poor performance.

**FIGURE 6:**
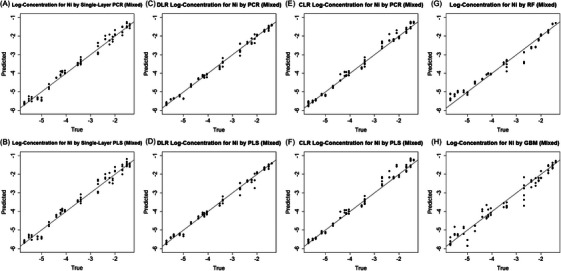
Predicted versus true log‐concentrations (mol L^−^^1^) for Ni in the mixed test set. Subpanels: (A) single‐layer principal component regression (PCR), (B) single‐layer partial least squares (PLS), (C) dynamical‐layer regression (DLR) PCR, (D) DLR PLS, (E) classified‐layer regression (CLR) PCR, (F) CLR PLS, (G) random forests, and (H) gradient boosting. The diagonal represents perfect prediction.

For Ni prediction, we also show the parameter tuning details in Figure S4 and Table S3. Both PCR and PLS achieve their best performance when s is about 4 in the DLR and C is 4 in the CLR. With the optimal parameters, DLR reduces the RMSE from 0.19 to 0.14 for PCR and from 0.21 to 0.15 for PLS, as shown in Table 3. With DLR, *R^2^* values increase above 0.99. CLR's results are mixed, reducing the RMSE to 0.18 for PCR but increasing it to 0.22 for PLS. From the scatter plots in Figure 6, we also find that DLR delivers the most notable improvement, particularly at the low and high ends of the log‐concentration range. CLR's mixed performance stems from reduced performance near class boundaries. The two global nonlinear models, RF and GBM, underperform relative to the single‐layer PCR and PLS, likely due to the moderate size of the training sample.

**TABLE 3:** Root mean squared error (RMSE) and *R^2^* comparison across single‐layer PCR/PLS, multi‐layer PCR/PLS (DLR and CLR), random forests (RFs), and gradient boosting machines (GBMs) for Ni log‐concentration prediction in the mixed NiSO_4_/CoSO_4_ dataset. Artificial neural networks (ANNs) excluded due to poor performance.

Method	PCR (Single)	PCR (DLR)	PCR (CLR)	PLS (Single)	PLS (DLR)	PLS (CLR)	RFs	GBMs
**RMSE**	**0.1922**	**0.1377**	**0.1799**	**0.2091**	**0.1452**	**0.2220**	**0.2695**	**0.3115**
*R^2^*	0.9824	0.9910	0.9847	0.9793	0.9900	0.9767	0.9657	0.9541

In contrast, predicting Co ion concentrations in the mixed dataset proves challenging for all models considered. In Table 4, PCR and PLS yield negative *R^2^* values, while RFs and GBMs achieve only moderate accuracy. Scatter plots in Figure S5 also demonstrate the poor prediction. Since no model can provide a reliable initial estimate, implementing an effective multi‐layer approach for predicting Co ion concentrations in the mixed dataset is infeasible. These limitations are attributed to spectral interference and the dominance of Ni‐related variance in the mixed spectra. From Figure 2, we find that the 300–400 and 500–750 nm regions of the spectra are dominated by Ni variance, whereas only the 400–500 nm region corresponds to Co. In Figure 3 for the mixed dataset, overlapping signals at 400–500 nm may lead to false Co responses at low concentrations, resulting in poor predictions across all models.

**TABLE 4:** Root mean squared error (RMSE) and *R^2^* for principal component regression (PCR), partial least squares (PLS), random forests (RFs), and gradient boosting machines (GBMs) for Co log‐concentration prediction in the mixed NiSO_4_/CoSO_4_ dataset; all models show limited accuracy due to spectral interference. Dynamical‐layer regression (DLR) and classified‐layer regression (CLR) are not applicable (see Section 3.4).

Method	PCR (Single)	PLS (Single)	RFs	GBMs
RMSE	1.6232	1.5678	1.1662	1.0007
*R^2^*	Negative	Negative	0.3008	0.4853

## 4 Conclusion

This study addresses the challenge of predicting wide‐range chemical concentrations from spectroscopic data, a task complicated by high dimensionality, multicollinearity, and nonlinearity. The proposed multi‐layer framework, comprising the DLR and CLR methods, represents a significant advancement in overcoming these challenges. Both methods improve predictive accuracy by narrowing the concentration range used to train local models: DLR employs dynamic layering, while CLR applies data classification.

Our experiments with single‐chemical datasets demonstrate that the multi‐layer framework effectively handles broad concentration ranges, as indicated by lower RMSE and higher *R* ^2^ values. The results also highlight the importance of parameter tuning for optimal model performance. In mixed‐chemical scenarios, the framework similarly improves prediction accuracy, provided that the initial rough estimate from the first‐layer model is reasonably reliable. Overall, this work contributes practical solutions for spectroscopic analysis and establishes a multi‐layer framework within chemometric methodologies. It suggests directions for future research on adapting machine learning methods for more complex spectral quantification settings.

## Author Contributions

**Muhammed ALjifri**, **Yanjun Qian**, and **Ye Chen** developed algorithms and wrote the main manuscript text; **Mo Jiang** designed experiments and provided supervision for **Carter Miller** to prepare the experiment and collect the data. **Yanjun Qian**, **Ye Chen**, and **Mo Jiang** reviewed the manuscript.

## Conflicts of Interest

The authors declare no conflicts of interest.

## Supporting information




**Supporting File**: ansa70078‐sup‐0001‐SuppMat.docx.

## Data Availability

The data that support the findings of this study are available from the corresponding author upon reasonable request.
