# Supplementary material for: Beyond Beer‐Lambert Linear Regression: Multi‐Layer Modeling for Wide‐Range Concentration Quantification Using Ultraviolet‐visible Spectroscopy
Source: Anal Sci Adv. 2026 Apr 2;7(1):e70078. doi: 10.1002/ansa.70078 (PMC13052029; doi:10.1002/ansa.70078)
Supplement: Supplementary file 1 — Supporting File: ansa70078‐sup‐0001‐SuppMat.docx. [file ANSA-7-e70078-s001.docx]

**Supplementary Material: Beyond Beer-Lambert Linear Regression: Multi-Layer Modeling for Wide-Range Concentration Quantification Using UV-Vis Spectroscopy**

Muhammed ALjifri^1^, Carter Miller^2^, Yanjun Qian^3,*^, Ye Chen^4^, Mo Jiang^2,5^

^1^Department of Mathematics, College of Science & Arts, King Abdulaziz University, Rabigh, Saudi Arabia

^2^Department of Chemical & Life Science Engineering, Virginia Commonwealth University, Richmond, USA

^3^Department of Statistical Sciences and Operations Research, Virginia Commonwealth University, Richmond, USA

^4^Schmidthorst College of Business, Bowling Green State University, Bowling Green, Ohio, USA

^5^The Polytechnic School, Arizona State University, Mesa, USA

^*^Corresponding author: [yqian3@vcu.edu](mailto:yqian3@vcu.edu)


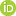
 [orcid.org/0000-0002-1581-0431](https://orcid.org/0000-0002-1581-0431)

# Tables

Table S1: RMSE and *R²* for single-layer, DLR, and CLR models using PCR and PLS for NiSO₄ log-concentration prediction with optimized parameters.

| Method | PCR | | | PLS | | | |
| --- | --- | --- | --- | --- | --- | --- | --- |
|  | Parameters | RMSE | *R²* | Parameters | RMSE | *R²* |  |
| Single-layer | nComp= 85 | 0*.*2912 | 0*.*9583 | nComp= 40 | 0*.*3097 | 0*.*9518 |  |
| DLR | *s* = 7*.*943 | 0*.*1534 | 0*.*9882 | *s* = 5*.*012 | 0*.*1463 | 0*.*9892 |  |
| CLR | *C* = 4 | 0*.*1852 | 0*.*9828 | *C* = 4 | 0*.*1625 | 0*.*9867 |  |

Table S2: RMSE and *R²* for single-layer, DLR, and CLR models using PCR and PLS for CoSO₄ log-concentration prediction with optimized parameters.

| Method | PCR | | | PLS | | |
| --- | --- | --- | --- | --- | --- | --- |
|  | Parameters | RMSE | *R²* | Parameters | RMSE | *R²* |
| Single-layer | nComp= 40 | 0*.*4304 | 0*.*9183 | nComp= 20 | 0*.*4941 | 0*.*8923 |
| DLR | *s* = 794*.*3 | 0*.*2430 | 0*.*9739 | *s* = 501*.*2 | 0*.*2382 | 0*.*9750 |
| CLR | *C* = 5 | 0*.*2686 | 0*.*9682 | *C* = 5 | 0*.*2888 | 0*.*9632 |

Table S3: RMSE and *R²* for single-layer, DLR, and CLR models using PCR and PLS for Ni log-concentration prediction in the mixed NiSO₄/CoSO₄ dataset with optimized parameters.

| Method | PCR | | | PLS | | |
| --- | --- | --- | --- | --- | --- | --- |
|  | Parameters | RMSE | *R²* | Parameters | RMSE | *R²* |
| Single-layer | nComp= 129 | 0*.*1922 | 0*.*9824 | nComp= 40 | 0*.*2091 | 0*.*9793 |
| DLR | *s* = 3*.*981 | 0*.*1377 | 0*.*9910 | *s* = 3*.*981 | 0*.*1452 | 0*.*9900 |
| CLR | *C* = 4 | 0*.*1799 | 0*.*9847 | *C* = 4 | 0*.*2220 | 0*.*9767 |

# Figures


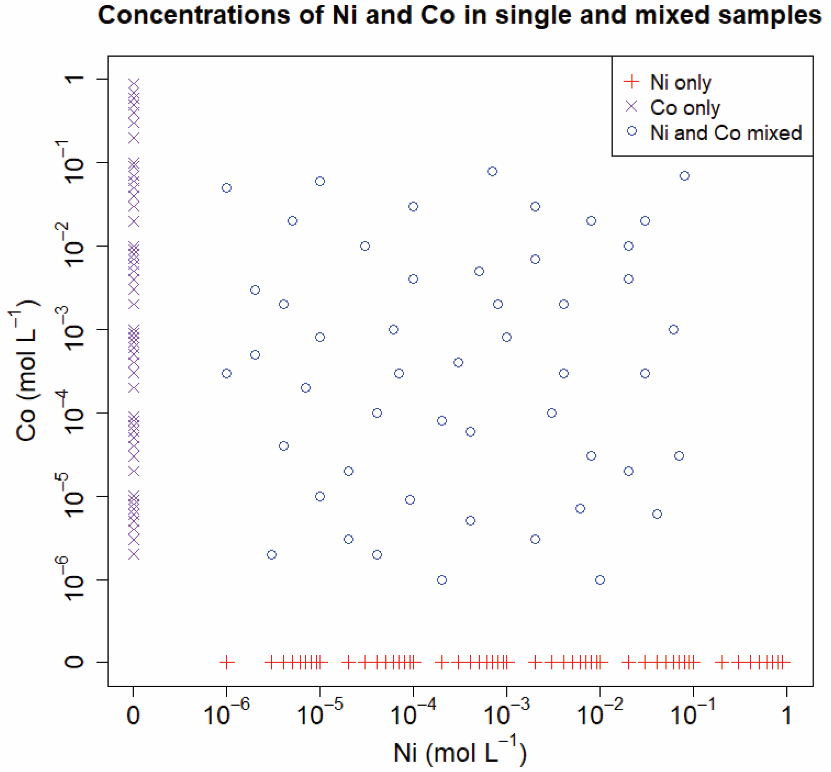


Figure S1: Molar concentrations of Ni and Co across all three experimental datasets on logarithmic axes. Red pluses: NiSO₄ single-chemical; purple crosses: CoSO₄ single-chemical; blue circles: mixed solutions generated via Latin hypercube sampling.


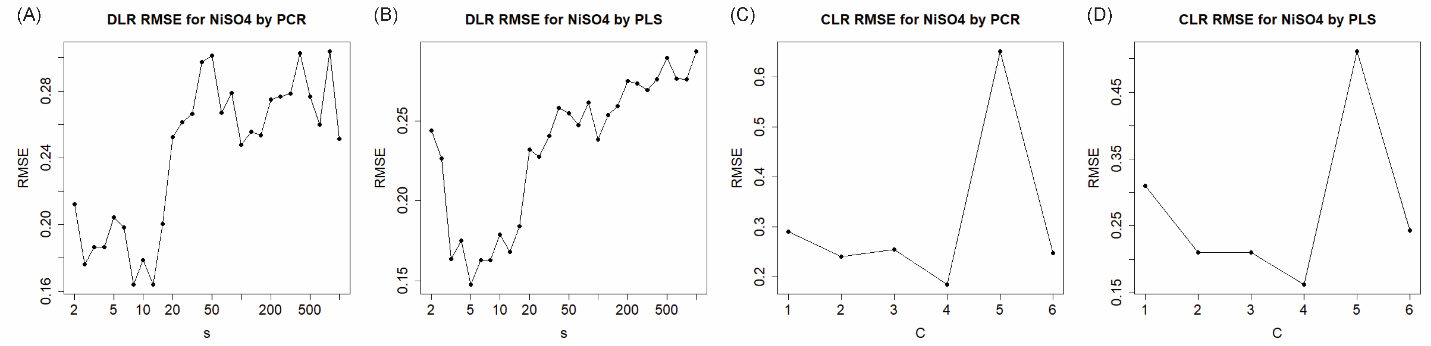


Figure S2: Change of RMSE when tuning the DLR scaling parameter s (A, B) and CLR class number C (C, D) for the NiSO₄ dataset, using PCR (A, C) and PLS (B, D). Optimal performance is achieved near *s* = 5–10 and *C* = 4.


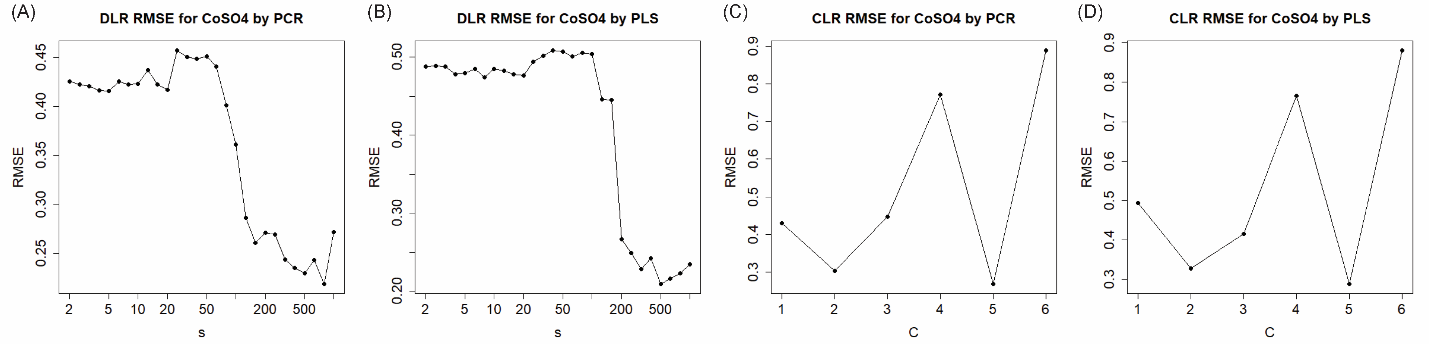


Figure S3: Change of RMSE when tuning the DLR scaling parameter s (A, B) and CLR class number C (C, D) for the CoSO₄ dataset, using PCR (A, C) and PLS (B, D). Optimal performance is achieved near *s* = 500–800 and *C* = 5.


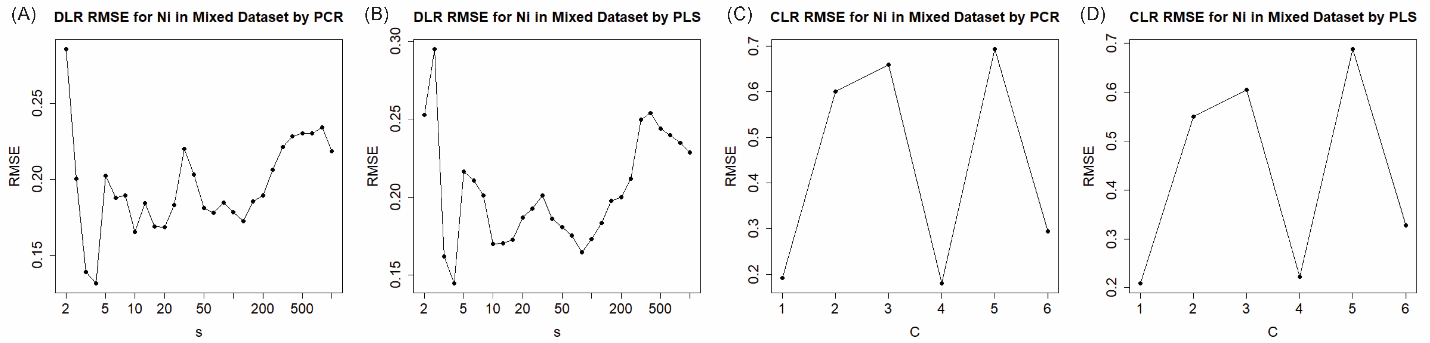


Figure S4: Change of RMSE when tuning the DLR scaling parameter s (A, B) and CLR class number C (C, D) for Ni prediction in the mixed dataset, using PCR (A, C) and PLS (B, D). Optimal performance is achieved at *s* about 4 and *C* = 4.


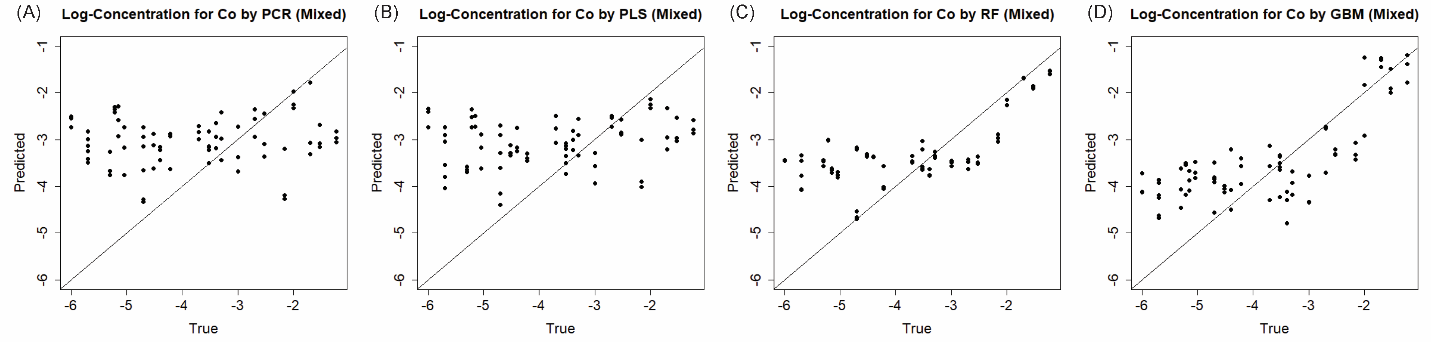


Figure S5: Predicted versus true log-concentrations (mol L⁻¹) for Co in the mixed test set using (A) PCR, (B) PLS, (C) random forests, and (D) gradient boosting. All models yield poor predictions due to spectral interference from Ni dominating the mixed-solution spectrum.
